# Supplementary figures and images for: Population-Specific Differences in Pathogenic Variants of Genes Associated with Monogenic Parkinson’s Disease
Source: Genes (Basel). 2025 Apr 15;16(4):454. doi: 10.3390/genes16040454 (PMC12027003; doi:10.3390/genes16040454)

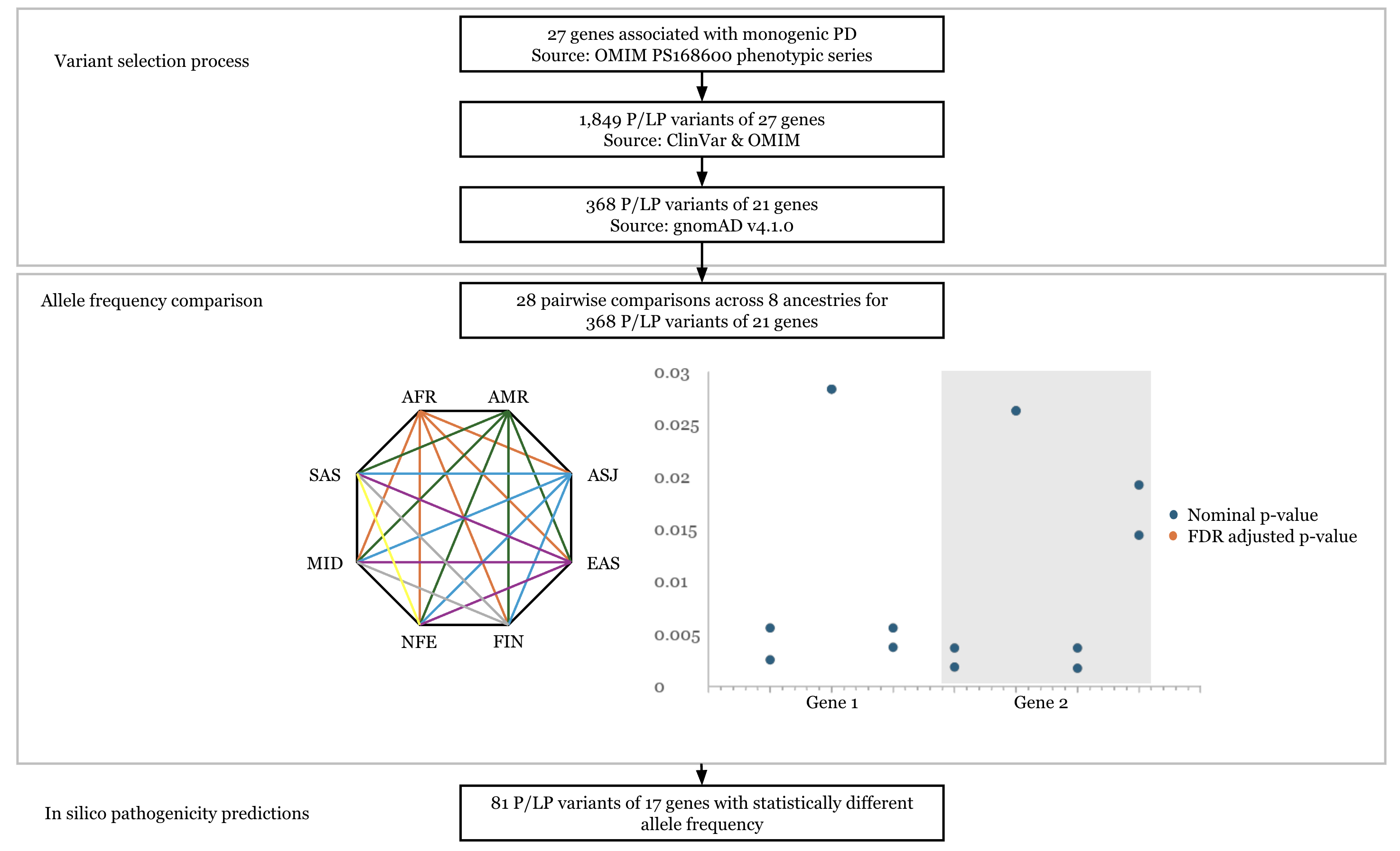

Supplement: Supplementary file 1 [file genes-16-00454-s001.zip › Supplementary Figure S1.png]
